# The Eulogium of Dr. Dake

**Published:** 1895-04

**Authors:** S. F. Shannon

**Affiliations:** The Denver Homœopathic Medical College and Hospital; Denver, Col.


### THE EULOGIUM OF DR. DAKE.

The Denver Homœopathic Medical
College and Hospital.
Denver, Col., March 27th, 1895.

Editor of The Homœopathic Physician :—It is something new for me to have any complaint to make against The Homœopathic Physician with regard to either quality or quantity, but I would be a traitor to the cause I espouse did I not enter my protest against the remarks made in the March number of the journal by Dr. Waggoner, of Larned, Kansas.

Having known Dr. J. P. Dake ever since I knew what a doctor was (he having been a neighbor to us in Pittsburg for many years), having listened to him as a Professor in my *Alma Mater* (Hahnemann, of Philadelphia), having always known him as a very dear friend of my parents and also of my dearest professional brethren in Pittsburg and also in Philadelphia,

I deny most emphatically Dr. Waggoner's statements that Dr. Dake was either an avowed heretic to Homœopathy, a traitor to Hahnemannian methods, or a pervertionist of materia medica.

I wish to do just the reverse to what Dr. Waggoner has done, and that is to enter my thanks to the journals that have so nobly come out and eulogized a man who was one of the grandest of the Homœopathic School.

Fraternally,

S. F. Shannon, M. D.

